# Exploring taught masters education for healthcare practitioners: a systematic review of literature

**DOI:** 10.1186/s12909-019-1768-7

**Published:** 2019-09-05

**Authors:** Mohammad Madi, Hayat Hamzeh, Mark Griffiths, Alison Rushton, Nicola R. Heneghan

**Affiliations:** 1 Department of Physical and Occupational therapy, Faculty of Applied Medical Sciences, Hashemite University, Zarqa, 13133 Jordan; 2 Department of Physiotherapy, School of Rehabilitation Sciences, The University of Jordan, Amman, 11942 Jordan; 3 School of Sport, Exercise and Rehabilitation Sciences, University of Birmingham, Birmingham, B15 2TT UK; 4 Centre of Precision Rehabilitation for Spinal Pain, School of Sport, Exercise and Rehabilitation Sciences, University of Birmingham, Birmingham, B15 2TT UK

**Keywords:** Masters education, Programme outcomes, Impact evaluation

## Abstract

**Background:**

Masters-level education is a key pathway of professional development for healthcare practitioners. Whilst there is evidence that Masters-level education leads to career enhancement, it is unclear how the programme pedagogy contributes to this. The objective was to: (1) examine the programme pedagogies and context that supports learning, and (2) synthesise the outputs, outcomes and impact of Masters-level healthcare programmes.

**Methods:**

A systematic review was conducted according to the Cochrane Collaboration handbook and is reported in line with PRISMA. Using pre-defined key terms and eligibility criteria, two reviewers independently searched Medline, ERIC, Web of Science, ProQuest, and CINAHL Plus databases from inception to 14th November 2016, reference lists of retrieved articles and selected websites. Data were extracted independently. The Mixed Methods Appraisal Tool was used to assess methodological quality. A Weight of Evidence Framework enabled evaluation of the overall quality of evidence. Data were synthesised using thematic qualitative analysis.

**Results:**

Thirty-five studies were included. All studies were retrospective, evaluated programmes in nursing (*n* = 19), physiotherapy (*n* = 6), general and family medicine (*n* = 4), public health (*n* = 3), dentistry (*n* = 1), interdisciplinary (*n* = 1), and occupational therapy (*n* = 1). Most studies were rated low in methodological quality, with an overall low to moderate weight of evidence for programmes’ outcomes and impact. Pedagogies that promote social participation and knowledge co-construction, reflection, learner-centred approach, relevance and authenticity influenced outcomes and impact.

**Conclusion(s):**

Notwithstanding the low to moderate weight of evidence, the review identified multiple positive outcomes of Master-level education for healthcare practitioners. Whilst the pedagogies that contributed to such positive outcomes were examined in some studies, there is a need to further explore links between programme pedagogy, outputs, outcomes and impact. A cultural approach to evaluation may capture how M-level education drives changes.

**Electronic supplementary material:**

The online version of this article (10.1186/s12909-019-1768-7) contains supplementary material, which is available to authorized users.

## Background

### Rationale

Preparing expert healthcare practitioners with a high level of competencies is an urgent global need to ensure the delivery of advanced level of patient care [1, 2]. To enable this, healthcare practitioners, those involved in the delivery of patient care, are required to engage in professional development activities [3] with many undertaking Masters’ level (M-level) education [4]. M-level education is defined for the purpose of this review as postgraduate formal and structured education based in higher education institution leading to either a Postgraduate Diploma or a Master of Science qualification. This level of education has been associated with an increase in the number of practitioners seeking senior or advanced practice roles [5, 6]. In the context of specialist M-level physiotherapy education, authors suggest that M-level education forms the basis for developing clinical expertise [7–11], although there is limited understanding of how the learning culture contributes to this; that is an understanding how interactions at the learners, programme and workplace levels supports change.

The learning culture of M-level education involves structured engagement with multiple pedagogies and contexts over a period of 1–2 years of full time study [12]. Understanding how this learning culture supports professional learning requires the use of logic model that maps out the programme’s outputs, outcomes, impact and the context of change [13–16]. This is rooted in a cultural view of learning which rejects the premise that learning is a process of acquiring and transferring knowledge [13]. Instead it is suggested that professional learning extends beyond the learning site and involves sociocultural contextual factors that modulate and shape a practitioner’s learning experience by influencing the interaction between a programme’s output, outcome, and impact [14–16]. Although such contextual factors are arguably external to programme activities [17], they are integral to a programme’s learning culture by virtue of the learners’ biographies [18]. It is less clear, however, how much M-level pedagogies draw on this cultural dimension of professional learning.

Three low to medium quality systematic reviews [19–21], rated using AMSTAR [22], have explored the influence of M-Level education in a healthcare context, with the most recent one including studies up to November 2011. Whilst these reviews explored the outcomes and impact of M-level programmes, there was limited exploration of programme pedagogies and contexts that drove changes. Moreover M-level education was not clearly defined which affected confidence in findings due to the inclusion of studies that evaluated pre-registration entry-level Masters and combined M-Level/PhD programmes. Therefore, conclusions were not specific to M-level education resulting in a need for a robust current systematic review that examines how M-level education in healthcare supports the professional development of practitioners.

### Objective

To undertake an evidence synthesis to explore how M-level education culture supports professional development of healthcare practitioners. In particular, to:

1. Understand the programme pedagogy and context that supports learning.
2. Identify the outputs, outcomes and impact of M-level education in supporting healthcare practitioners.

## Methods

A systematic review designed using the guidelines of the Cochrane Collaboration handbook was conducted [23]. The review is reported in line with Preferred Reporting Items for Systematic reviews and Meta-Analysis (PRISMA) guidelines [24].

### Eligibility criteria

Was informed by the adapted search concept tool ‘PICOS’ to ensure the objectives of the review could be achieved. In this instance Population referred to participants/graduates of M-level education, Intervention was M-level education, Outcome referred to programme outputs and impact and all forms of study design considered.

### Inclusion criteria

1. Studies that evaluated M-level healthcare programmes were included, specifically including Postgraduate diploma and Master of Science programmes.
2. Qualitative, quantitative and mixed-methods research designs.
3. Studies that were published in the English language.

### Exclusion criteria

1. Theoretical studies with no data collection
2. Evaluated online, long distance M-level courses.
3. Residency and Fellowship programmes that are not based in higher education settings

### Information sources and search strategy

Two independent reviewers (MM/HH) searched:

- Medline (Ovid), ERIC, Web of Science, ProQuest, and CINAHL Plus databases from inception to 14th November 2016.
- Reference lists of retrieved articles, websites (Google scholar, science direct, and Taylor and Francis) and grey literature (dissertations and theses)

The following is the search strategy used in Medline (Ovid) from 1946 until 14th November 2016:

1. Postgraduate education.mp.
2. Master’s level education.mp.
3. masters programme.mp.
4. Masters degree.mp.
5. professional development.mp.
6. 1 or 2 or 3 or 4 or 5
7. Evaluation.mp. or Evaluation Studies as Topic/
8. Impact.mp.
9. Outcome.mp.
10. output.mp.
11. 7 or 8 or 9 or 10
12. 6 and 11

### Study selection

Reviewers independently evaluated retrieved studies against the pre-specified eligibility criteria, rating each study as ‘eligible’, ‘not eligible’ or ‘might be eligible’. A third reviewer was available to mediate disagreements.

### Data extraction

Following piloting, one reviewer (MM) extracted data using an adapted Cochrane Collaboration’s data extraction form [23]. The second reviewer (HH) checked extracted data for accuracy. Data that answer the main review questions or aid assessing the quality of individual studies were extracted. These included: reference details, country, funding source, conflicts of interest, programme level, programme title, programme aims, study design, study aims, outcome measures, participants, method/s of recruitment, response rate, obtained consent, ethical approval, programme activities and pedagogy, point of approaching graduates, evaluation model used, programme outputs, outcomes, and impact. Authors of eligible studies were contacted to retrieve missing or clarify ambiguous data.

To ensure precise and consistent data extraction, the Logic Model [25] definitions of programme output, outcome, and impact were used. The Logic Model has previously been used to map programme design, structure, output, outcome, and impact [26]. Where output is used to refer to the direct products of programme activities which may include types, levels and targets of services to be delivered by the programme; outcomes describe the specific changes in participants’ behaviour, knowledge, skills, status and level of functioning; and impact refers to the fundamental intended or unintended change occurring in organizations, communities or systems as a result of programme activities within 7 to 10 years [25, 27]. Thus, the use of these terms offered a unified lens to review M-level programme evaluation literature.

### Methodological quality assessment

The two reviewers (MM/HH) independently assessed the methodological quality of included studies. The Mixed Method Appraisal Tool (MMAT) was used [28]. MMAT is a valid (based on experts judgment), reliable (ICC = 0.80) and efficient tool to critically appraise the methodological quality of studies of differing designs [28–30]. Additionally, a Weight of Evidence (WoE) Framework [31] was used to appraise the quality and relevance of evidence using pre-specified criteria of three domains: A) soundness of studies; B) appropriateness of study design for answering the review questions; and C) relevance of the study focus to the review. High WoE was defined as A) scoring more than 50% using MMAT, B) Drawing on multiple cohorts of students using a longitudinal pre-post study design, and C) defining primary outcomes with clear description of specific programme pedagogy. Medium WoE was defined as A) scoring 50% using MMAT, B) drawing on one cohort of learners using a longitudinal pre-post design, and C) defining primary outcomes with no specific description of programme pedagogy. Finally, low WoE was defined as A) scoring less than 50% using MMAT, B) a post hoc programme evaluation, and C) the lack of defined outcomes as well as proper description of programme pedagogy.

### Synthesis of results

The extracted data were tabulated and synthesised using thematic qualitative analysis [32]. Data were deductively coded using the logic model terminologies under the themes of programme pedagogy, contextual factors, outputs, outcomes, and impact. With iterative examination of data, each category under these main themes was clearly defined to establish its properties (Table 1). This was followed by capturing the relationships between coded data of various programmes in a way that illuminates the chain of reasoning of how and why they work. This analytical approach allowed mapping of M-level programme Logic Model across multiple healthcare disciplines.

**Table 1:** Synthesis of M-level education outputs, outcomes and impact

		Description	Data Source
Outputs	Successful collaborative work and student’s engagement	Successful formation and support of learning groups that facilitate collaborative peer interaction	[33–35]
	Perceiving relevance	The perception of relevance to practice leads to engagement	[36–38]
	Deconstructing knowledge	Questioning the effectiveness of practice and level of criticality that leads to reconstruction of M-level knowledge	[36, 39–42]
Outcomes	High level critical thinking skills and/or analysis	Locate and understand arguments, relationships, make sound inferences, and warranted conclusions.	[6, 34, 36, 38, 40, 43–50]
	High level clinical reasoning skills	Context-bounded cognitive processes used for clinical decision-making that draw on advanced level of knowledge	[6, 34, 36, 40, 42, 44, 46, 50–53]
	High confidence and motivation to practice	Developing senses of efficacy and advocacy that motivate graduates for clinical practice	[6, 34, 38, 41, 44, 47, 54, 55]
	High level communication skills	Effective communication with patients, colleagues, and other healthcare graduates	[33, 36, 44, 49, 52, 56, 57]
	Becoming lifelong learner	Motivation for professional development and learning from practice	[36, 39, 43, 44, 47, 48, 52, 58]
	Enhanced sense of autonomy	Ability to function without direct support	[36, 42, 43, 52]
	Enhanced career progression	Getting promoted or movement to advanced level career	[6, 38, 39, 41, 44, 47, 49, 54, 57, 59–62]
Impact	Management complex patient presentation	Understanding complex patient presentation, creative non-routine practice, understanding healthcare system, and demonstrating flexibility in role choices	[42, 45, 56, 60, 63]
	Assuming research, leadership and management positions	Driving changes in practice and service delivery and supporting clinical-based research	[56, 57, 64]
	Assuming teaching roles	Collegial teaching duties, supporting peer’s learning, and involvement in university education	[35, 36, 41, 47, 50, 52, 64, 65]
	Reduced direct patient care	Assuming more managerial, research, and teaching duties at the expense of direct patient care	[6, 43]
	Increased retention rate	Increased motivation to stay in clinical practice	[35, 44, 50]
	Patient Care	Describing change to direct patient care routine like earlier recovery and ability to self-manage	[49, 52, 65, 66]

## Results

### Summary and characteristics of included studies

**Fig. 1:**
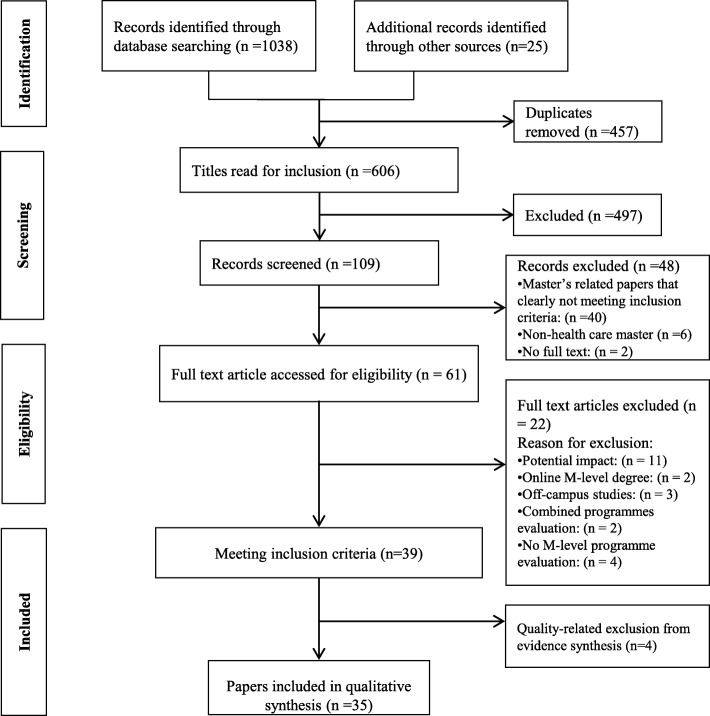
PRISMA Flow Diagram: Study selection process [24]

Thirty-five eligible studies that drew on the accounts of 2834 graduates and a total of 87 programme educators, clinical managers and workplace colleagues were included. See Fig. 1.

The characteristics of these studies are shown in Table 2. A list of excluded studies and reasons for exclusion are included in Additional file 1. Included studies explored M-level programmes originated from the UK (*n* = 16), USA (*n* = 3), Australia (*n* = 4), Ireland (*n* = 3), New Zealand (*n* = 3), Canada (*n* = 2), Jordan (*n* = 1), and Vietnam (*n* = 1). Two studies [60, 66] explored M-level programmes originated from multiple countries. All studies were retrospective in nature, and the exact timing of approaching graduates was unclear in 18 studies (Table 2).

**Table 2:** Characteristics of included studies

Reference	Study aim	Country	Specialty	Study design	Methods	Population	Time of approaching participants
Barnhill et al. [49]	Investigate impact on clinical practice	New Zealand	Nursing	Quantitative: descriptive	Postal survey	Registered nurses (*n* = 27) / Senior nurses (manager & educator) (*n* = 23) / *RR* = 47.7%	One year after
Baron et al. [35]	Investigate effects on career development	UK	General Practice	Quantitative: Descriptive	Questionnaire with open ended questions	GP graduates from 1997 to 2003 / A total of 150 questionnaires were sent out and 81 were returned (*RR* = 54%) / Total population is not stated	Between 2 and 6 years
Bearn and Chadwick [37]	Evaluate students’ experiences	UK	Orthodontic	Qualitative	Focus groups and semi-structured interviews	12 postgraduate students / First cohort of the programme	Directly after
Calvert and Britten [33] Calvert and Britten [34]	Exploring outcomes on professional and personal development	UK	General Practice	Qualitative	Free writing feedback	71 of 76 graduates from the first 9 cohorts (*RR* = 93%)	Unclear
Chaboyer and Retsas [38]	Evaluate programme outcomes	Australia	Nursing	Mixed: concurrent	Questionnaires consisted of open- and closed-ended questions	44 graduates out of 50 (88%) in 1st survey 37 graduates in second survey (77%) of the 1994 cohort Stakeholder	Directly after
Conneeley [39]	Evaluate students’ experiences, perceived benefits and impact on career.	UK	Occupational Therapy	Qualitative: Phenomenology	Focus group	Six students: 4 OT and 2 PT [total programme cohort]	Directly after
Constantine and Carpenter [36]	Explore practitioners’ development	UK	Physiotherapy	Qualitative: Phenomenology	Semi-structured phone interview	7 out of 35 invited alumni Total population is not stated – graduates from other programmes have been included	Unclear
Cragg and Andrusyszyn [63] Cragg and Andrusyszyn [59]	Identify perceived changes at personal, practice, and attitudinal levels	Canada	Nursing	Qualitative: descriptive	Semi-structured Face-to-face or telephone interviews	22 graduates who completed programs from 2000 to 2003 Total population: not stated	Directly after
Drennan [54]	Evaluate career and academic development	Ireland	Nursing	Quantitative: descriptive	Cross-sectional postal survey	220 out of 322 approached graduated between 2000 and 2005 (*RR* = 68%)	between 2 and 6 years
Drennan [48]	Measure critical thinking ability	Ireland	Nursing	Quantitative: cross sectional analytic	Watson–Glaser Critical Thinking Appraisal tool administered to two groups	Two cohorts: 83 of 110 students (75%) commencing MSc in Nursing programmes. & 140 of 222 students (63%) who had a MSc degree in Nursing between 2003 and 2007	Unclear
Drennan [56]	Evaluate graduates’ ability to change practice	Ireland	Nursing	Quantitative: cross-sectional survey	Retrospective pre-test design	140 of 222 students (63%) who had a MSc degree in Nursing	Directly after
Gerstel et al. [63]	Evaluate graduates’ competencies and career development	International	Public Health	Quantitative:	Online survey	177 of 327 invited alumni (*RR* = 54%)	between 2 and 6 years
Green et al. [6]	Identify the influence on career development	UK	Physiotherapy	Quantitative: descriptive Qualitative: descriptive	Postal survey & Focus group	Graduates from the MSc MSK PT programmes from 1994 to 2005 48 of 77 (*RR* = 62.3%)	Unclear
Perry et al. [41]	Explore impact on professional and personal development	UK	Physiotherapy	Qualitative: atheoretical pragmatic utilised within an interpretivist paradigm	Focus group	Seven graduates out of 11 agreed to take part. Pooled form respondent of Green et al. (2008)	Unclear
Le et al. [62]	Explore the relevance and impact on work	Vietnam	Public Health	Quantitative: descriptive Qualitative: descriptive	Postal survey Interviews	148 graduates out of the total of 187 (*RR* = 79.1%)	Unclear
LeCount [51]	Describe programme, inception, implementation, and outcomes.	USA	Nursing Geriatrics	Quantitative: descriptive	Postal survey	16 of 20 contacted (*RR* = 80%) / Total population: 30 graduates	Directly after
Murray et al. [61]	Analyse graduates’ satisfaction and explore the perceived impact.	USA	Interdisciplinary	Quantitative: descriptive	Postal questionnaire	53 of 85 graduates contacted / (*RR* = 62%) Total population: 96 graduates between 1982 and 1998 / 29 of 37 contacted employers (*RR* = 78%)	Unclear
Nicolson et al. [42]	Identify educational and working experiences	UK	Nursing	Mixed Methods: Sequential	Focus group that informed the Postal questionnaire & Semi-structured telephone interviews	Five cohorts of graduates Programme team nursing and medical staff at one NICU 37 questionnaires (*RR* = 71.2%)	Unclear
Pelletier et al. [55]	Investigate effects on practice and career	Australia	Nursing	Quantitative: descriptive	Postal questionnaire	40 of 55 from 1991 cohort. *RR* = 72% Total population: not clear	Directly after
Pelletier et al. [52]	Investigate the impact on patient care	Australia	Nursing	Quantitative: descriptive	Postal questionnaire	236 from Pelletier et al., (1998) / retention rate of 58% / Five cohorts	Between 2 and 6 years
Pelletier et al. [66]	Report motivators and barriers to career change	Australia	Nursing	Quantitative: Longitudinal descriptive and co-relational	Postal questionnaire	151 of 236 in Pelletier et al., (2003)	Between 7 and 10 years
Petty et al. [40] Petty et al. [58]	Describe impact clinical practice Develop an explanatory theory of the learning transition	UK	Physiotherapy	Qualitative: grounded theory. Theory-seeking case study	Semi-structured interviews	11 alumni agreed of 35 purposefully selected	Between 2 and 6 years
Spence [45] Spence [46]	Evaluate the clinical impact	New Zealand	Nursing	Qualitative: descriptive	Loose-structured interviews	12 graduates of a clinically focused programme. 8 co-worker and/or employer	Unclear
Spencer [43]	Examine impact on professional practice	UK	Nursing midwives and health visitors	Qualitative: Phenomenology	Semi-structured interviews	12 qualified nurses, midwives and health visitors since its inception in 1998	Unclear
Stark [60]	Investigate differences in role choices, role flexibility, and practice settings	USA	Nursing	Quantitative: cross-sectional, comparative study	Postal survey: self-report Role Choices, Role Flexibility, and Practice Settings Survey	406 of 1086 potential (*RR* = 37.4%) Final completed questionnaires: 285 Power analysis: 165 required	Unclear
Stathopoulos and Harrison [44]	Explore impact on professional practice	UK	Physiotherapy	Qualitative: Phenomenology	Focus group	5 of 7 graduates agreed to participate. Working in clinical setting	Between 2 and 6 years
Tsimtsiou et al. [50]	Assess benefits on professional and career development	UK	General Practice	Mixed	Postal questionnaire that include free writing	Graduates from 1997 until 2008 50 of 66 (*RR* = 76%)	Unclear
Whyte et al. [47]	Evaluate the professional relevance and the personal and career growth	UK	Nursing	Quantitative: descriptive	A self-administered questionnaire	Graduates from 1991 to 1994 109 of 190 posted questionnaire (*RR* = 57%)	Unclear
Wildman et al. [53]	Evaluate the effect on clinical practice.	UK	Nursing	Mixed	Postal questionnaire	The first seven cohorts of the programme (n:169) (*RR* = 66.8% (113))	Unclear
Zahran [65]	Explore motivational factors and explore perceived impact on practice	Jordan	Nursing	Qualitative: Ethnography	Semi-structured interviews	44 M-Level qualified nurses nurse educationalists clinical nurse supervisors	Unclear
Zwanikken et al. [57]	Examine the influence on performance at the workplace, and professional contribution to society	International	Public Health	Quantitative: descriptive	Self-administered questionnaire	*n* = 445 *RR* = 37.5%	Unclear

*RR* Response Rate, *GP* General Practitioner, *OT* Occupational Therapist, *PT* Physiotherapist or Physical Therapy, *NICU* Neonatal Intensive Care Unit

These studies drew on qualitative (*n* = 15), quantitative (*n* = 14) and combined qualitative and quantitative methods of data collection (*n* = 6) (Table 3). Most quantitative studies were descriptive, with only two cross-sectional analytical studies [51, 63] that compared M-level participant-students’ cohorts with other cohorts. Three studies used large scale alumni surveys to study career pathways in nursing [57], physiotherapy [6], and public health [66].

**Table 3:** Methods used in evaluating M-level education

Qualitative (*n* = 15)	Semi-structured interviews (*n* = 6): [36, 40, 43, 58, 59, 63]
	Graduates free writing (*n* = 2): [33, 34]
	Focus group (*n* = 3): [39, 41, 43]
	Graduates, Managers, Educators, and Colleague’s interviews (*n* = 3): [45, 46, 65]
	Focus groups and semi-structured interviews (*n* = 1): [37]
Quantitative (*n* = 14)	Graduates Survey (*n* = 10): [35, 47, 51, 52, 54–57, 64, 66]
	Cross sectional analytic (*n* = 2): [48, 60]
	Graduates, Managers, and Educators Surveys (*n* = 2): [60, 61]
Combined data collection (*n* = 6)	Graduates’ open- and closed-ended questionnaire (*n* = 3): [50, 53, 62]
	Graduates’ open- and closed-ended questionnaire, and stakeholders’ interviews (*n* = 1): [38]
	Graduates survey and focus group (*n* = 1): [6]
	Graduates survey, interviews and focus group (*n* = 1): [42]

## Methodological quality

Details of methodological quality of individual studies are described in Table 4. The methodological quality ranged from low to medium for most studies. Only four studies [40, 50, 54, 58] were deemed to be of high quality.

**Table 4:** Scores of Mixed Methods Appraisal Tool (MMAT), and Overall Weight of Evidence (WoE)

Reference	MMAT Score	WoE A	WoE B	WoE C	Overall WoE
Calvert and Britten [33] Calvert and Britten [34]	25%	Low	Low	Medium	Low
Baron et al. [35]	25%	Low	Low	Low	Low
Barnhill et al. [49]	25%	Low	Low	Low	Low
Bearn and Chadwick [37]	25%	Low	Low	Low	Low
Chaboyer and Retsas [38]	25%	Low	Low	Low	Low
Conneeley [39]	50%	Medium	Low	Low	Low
Constantine and Carpenter [36]	25%	Low	Low	Medium	Low
Cragg and Andrusyszyn [63] Cragg and Andrusyszyn [59]	50%	Medium	Low	Medium	Medium
Drennan [54]	75%	High	Low	Low	Medium
Drennan [48]	50%	Medium	Low	High	Medium
Drennan [56]	25%	Low	Low	Low	Low
Green et al. [6]	25%	Low	Low	Low	Low
Gerstel et al. [64]	25%	Low	Low	Low	Low
Le et al. [62]	50%	Medium	Low	Low	Low
LeCount [51]	25%	Low	Low	Low	Low
Murray et al. [61]	50%	Medium	Low	Low	Low
Nicolson et al. [42]	50%	Medium	Low	Low	Low
Pelletier et al. [55]	25%	Low	Low	Low	Low
Pelletier et al. [52]	50%	Medium	Low	Medium	Medium
Pelletier et al. [66]	25%	Low	High	Low	Medium
Perry et al. [41]	50%	Medium	Low	Medium	Medium
Petty et al. [40] Petty et al. [58]	75%	High	Low	Medium	Medium
Spence [45] Spence [46]	50%	Medium	Low	Medium	Medium
Spencer [43]	25%	Low	Low	Low	Low
Stark [60]	50%	Medium	Low	Low	Low
Stathopoulos and Harrison [44]	25%	Low	Low	Low	Low
Tsimtsiou et al. [50]	75%	High	Low	Low	Medium
Whyte et al. [47]	25%	Low	Low	Low	Low
Wildman et al. [53]	25%	Low	Low	Medium	Low
Zahran [65]	25%	Low	Low	Low	Low
Zwanikken et al. [57]	25%	Low	Low	Medium	Low

### Logic model synthesis

**Fig. 2:**
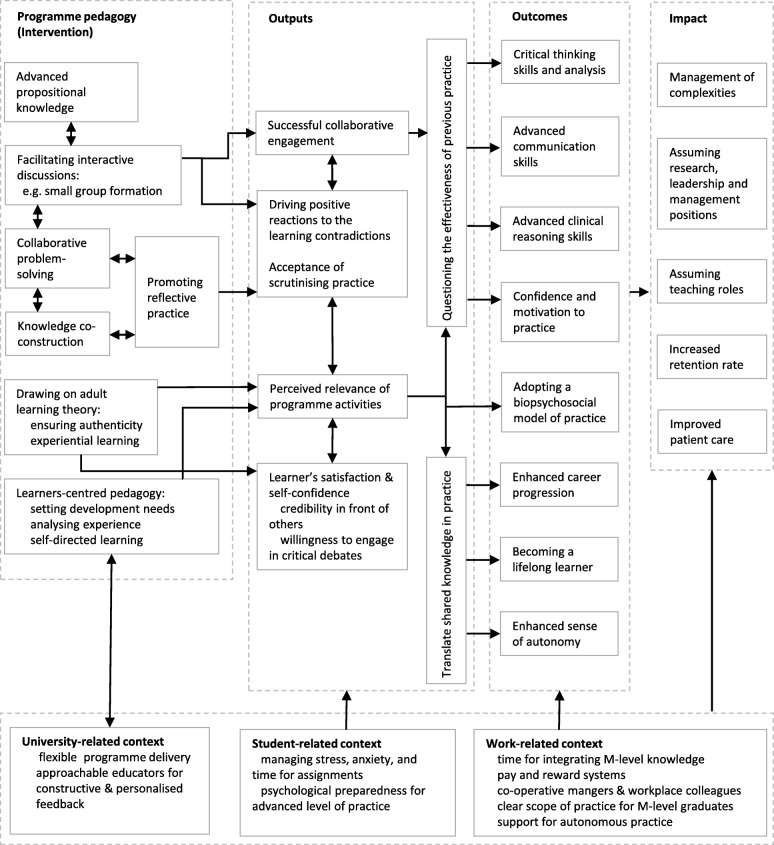
M-level education logic model synthesised from systematic review of literature. (Note: Whilst it is read from left to right, the pathway does not imply causality)

The synthesis of the findings into a completed programme theory Logic Model is illustrated in Fig. 2. This synthesis of M-level programme theory across several healthcare professions offers a pathway that represents how the programme pedagogical activities and context interacted to produce outputs, outcomes and impact. The inclusion of programme activities and context by some studies facilitated the collective synthesis of this model.

### Programme pedagogy

Three studies reported a programme’s modules with no further exploration of its pedagogy [36, 53, 64]. The pedagogies of nine programme evaluations were described (Additional file 2). These pedagogical approaches were synthesised into five categories. They were generally informed by social constructivism, adult learning, and reflective learning theories, with an overall structure that promoted a learner centred approach. The participant-students’ perceptions of a programme’s pedagogy were not explored.

#### The role of theoretical content

Graduates in five studies [38, 40, 42, 53, 58] explicitly referred to the role of a programme’s theoretical component as a source of advancing practice. They believed that the specialist theoretical knowledge they received positively influenced their professional development, particularly where the theoretical aspect of education was not covered in undergraduate education, leading to a belief that incorporating, or lack of, propositional knowledge in M-level education can directly impact the overall advancement of graduate’s skills.

#### Social participation and knowledge co-construction

Across physiotherapy, nursing, general practice, and public health programmes, social learning supported peer-peer communication and co-construction of knowledge and experiences [34, 35, 38], especially when done in small groups [35]. The collaborative and problem-solving environments were valued by graduates when compared to their pre-masters rote-learning educational experiences [44, 47]. Social learning was thought to promote integration of the shared knowledge and experiences in clinical practice, and adoption of a biopsychosocial model of practice [63].

#### Environment for reflection

A few researchers found that reflection on experience helped the most in advancing clinical reasoning skills [35, 38, 58]; especially when students documented processes of reflection [33]. However, no study reported the processes of reflection experienced by graduates. In contrast, learning transition occurred when educators facilitated students’ critical reflections and provided feedback on performance [40].

#### Learner-centred approach

Graduates of multiple programmes believed that adopting a learner-centred pedagogy contributed to positive outcomes [35, 47, 48]. The learner-centred pedagogy included setting out learning and development needs [35]; flexibility of the programme delivery [47]; analysing progress throughout the programme [35]; and encouraging students to speak their mind during interactive discussions [48].

#### Drawing on adult learning theory

Three studies indicated that programmes drew on principles of adult learning theory [35, 61, 63]. Promoting self-directed learning was the most prominent feature, in terms of graduates assuming responsibility for identifying personal and professional development needs [44].

### Programme outputs component of the logic model

Programme outputs are the direct product of programme activities which facilitate achieving programme outcomes [25]; and include participant-students’ reactions to programme activities [25]. Three main outputs were sporadically documented in 10 studies of low to medium quality (Table 1). These outputs highlight the importance of changing learner’s attitudes in order to drive transformative changes.

#### Successful collaborative work

While successful formation and support of learning groups, including small class size and promoting diversity of opinions drove learning engagement in some cohorts [34, 36, 44], poor attendance for some students created a sense of frustration which affected group dynamics [35].

#### Relevance of programme activities

Ensuring the relevance of programme activities to students’ clinical practice cultivated greater satisfaction and engagement which invariably led to achievement of programme outcomes [36–38].

#### Positive reactions to the learning contradictions

Achieving learning outcomes was found to be contingent on the students’ positive reactions to the learning contradictions that characterise M-level education [36, 39, 41, 44, 50, 58]. Graduates of these programmes suggested that achieving programme outcomes was associated with questioning the effectiveness of their previous practice, which lead to a process of reconstruction of their knowledge and skills. This process was described as ‘shrugging off the old’ and ‘assuming the new’ [50].

### Programme outcomes component of the logic model

Seven main outcomes of M-level education were reported in 22 studies (Table 1). These outcomes reflected changes in graduates’ behaviour, knowledge, skills, status and level of functioning. They ranged from advancement of critical thinking and clinical reasoning skills to an enhanced career progression for most graduates.

#### Critical thinking skills and analysis

Advancement of critical thinking skills was identified across all healthcare disciplines. Significant differences in critical thinking ability between graduates and freshmen of six nursing programmes in Ireland were identified using the Watson–Glaser Critical Thinking Appraisal tool [48]. The remaining evidence of advancement in critical thinking resulted from qualitative research. Graduates demonstrated a transformation from non-critical, routine and therapist-centred practice to a more critical and patient-centred one [40]. Participating in M-level education advanced graduates’ abilities to critically discuss research evidence [34, 36, 47] which enabled them to justify their own practices [43, 58]. In one study of acute nurse graduates, their perceptions of advancement in critical thinking skills was not associated with a tangible effect on patient care such as length of hospital stay [49]. Moreover, some students questioned their ability to continue at this level of high criticality upon returning to their workplace environment [39].

#### Clinical reasoning skills

M-level education advanced graduates’ clinical reasoning skills during both assessment and treatment phases of patient management [36]. This was associated with open mindedness in selecting alternative management options [58]. Likewise, graduates became more attentive to details, able to interpret patient data, and articulate diagnostic and treatment decisions [46]. While there was limited exploration of how programme pedagogy supported change, some studies attributed it to the theoretical aspect of the curriculum. For example, graduates of some programmes demonstrated an advanced understanding of ethical reasoning because of a module or content that is related to ethical issues [50, 52, 53]. Also, a cohort of physiotherapy graduates suggested that the limited psychosocial content of their curriculum adversely affected their ability to manage patients with complex psychosocial issues [58].

#### Confidence and motivation to practice

Increased confidence and motivation in clinical practice was described by graduates of several programmes (Table 1). In particular, changes involved increased credibility in front of others [44]; increased willingness to engage in critical debates [34]; enhanced ability to conduct and publish scholarly research [47]; and enhanced capabilities to meet the requirements of extended scope practice, clinical specialist, and consultant roles [6, 43]. The evidence suggests that confidence improved as a result of the specific professional knowledge provided at this level of education [38, 58]. The perceived sense of efficacy allowed graduates to advocate practice and policy changes and to support professional learning of junior colleagues [41, 44, 52].

#### Enhanced career progression

Some studies reported, but poorly defined, a career progression as an outcome of M-level education. For example, 84% of nurse graduates agreed or strongly agreed that their programme promoted career progression through promotion, increased payment, and change of job description or specialisation [60]. For some graduates, career changes occurred either during or upon completion of the programme, indicating a high demand on M-level qualified practitioners [6, 38, 39]. What is less clear, however, is the impact of career progression on direct patient care since graduates from some programmes assumed management, research and education duties [6, 47].

#### Becoming a lifelong learner

M-level education supported engagement in a lifelong learning process [36, 39, 43, 47]. It provided graduates with the tools to “learn how to learn” [39], and become an adult learner [36]. However, such tools and processes were poorly defined. On the other hand, graduates were thought to be able to learn from their practice though processes of reflection [58]. Therefore, becoming a lifelong learner was not only limited to locating sources of knowledge, but also extended to synthesising practice-based knowledge, which was linked to patient-centred practice, ongoing introspection and self-critique [48, 53].

#### Advanced communication skills

Graduates in public health reported a 78% increase in communication competencies [57]. Multiple areas of advancement were identified in nursing cohorts including: oral communication, written communication, working and coping with team conflicts, understanding team members’ feelings, listening to others, and communication with colleagues [56]. Three studies [33, 50, 52] reported advancement in communication with patients, however two studies [33, 50] reported that this was the least developed outcome.

#### Enhanced sense of autonomy

Whilst an increase in graduates’ autonomy was reported in multiple studies [36, 43, 50, 52], the authors did not to evaluate what this meant or entailed. Seventy-five percent of M-level nurse graduates (*N* = 236) reported an increase in ability to assume work roles independently [52]. The authors attributed this to the enhanced level of self-confidence [36, 52]. However, they argued that 22% of graduates who did not perceive an increase in autonomy were working within a healthcare system that does not support autonomous practice [36, 52], something that was also identified in another study [50] leading the authors to believe that workplace context could have an adverse effect on M-level graduates’ professional development.

### Programme impact component of the logic model

Programme impact is conceptualised as the fundamental intended or unintended change occurring in organizations, communities or systems within 7 to 10 years after the programme [25]. Due to the variability between the time of graduation and the time of approaching the graduates (Table 2), the synthesis of the evidence of programme impact is limited. One study [57] clearly stated that they approached participant 7–10 years after the programme, although for many this was unclear. Impact was self-reported in most studies (Table 1), with two studies [49, 65] drawing on stakeholders such as managers and workplace colleagues. The main domains of M-level programme impact were: management of complexities; assuming research, leadership and management positions; assuming teaching roles; increased retention rate of practitioners; and enhanced patient care.

#### Management of complexities

M-level education enhanced graduates’ abilities to understand the healthcare system [56, 63], demonstrate flexibility in management decisions [58, 60], and demonstrate creative practice [45]. Moreover, graduates were able to manage complex patient presentations [50]. They also showed attitudes of appreciating others’ perspectives, thinking analytically, defining problems, and resolving conflicts [56].

#### Assuming research, leadership and management positions

Data suggests that M-level education enhanced graduates’ research, leadership and management skills putting them in a position to drive change in practice and service delivery [56, 57]. M-level public health graduates were able to evaluate service delivery and recommend development needs [57], however, it was not clear whether these changes were at a local level i.e. graduate’s workplace or at a national level.

#### Assuming teaching roles

Engagement in collegial teaching duties, supporting a peer’s learning, and involvement in university education were reported in multiple evaluations (Table 1). Involvement in teaching activities was not only an opportunity for M-level graduates to give back to society, but also an opportunity for them to engage in a lifelong learning process [36, 40, 48, 52].

#### Increased retention rate of practitioners

In one study, graduates expressed a tendency to remain in clinical practice because of increased motivation and confidence [35]. This was evident in the cases of practitioners with more experience because of the fresh perspectives and insights offered by M-level education [50], which led some graduates to express their desires to implement knowledge and skills within the clinical context [44]. In the UK context, graduate retention was associated with the presence of clear National Health Service (NHS) scope of practice that acknowledges and rewards M-level graduates [6].

#### Patient care

The data demonstrated limited exploration of the impact of M-level education in terms of capturing tangible changes to patient care. For example, duration of recovery. It was implicitly demonstrated that indirect improvement of the quality of patient care is plausible through advancement in knowledge, cognitive and clinical reasoning skills [58], particularly in terms of embracing patient-centred practice [50, 53]. Nonetheless, some managers or colleagues of nursing programme graduates did not perceive any change in direct patient care [49, 65]. In one study, this was attributed to workplace restrictions or practice policies that do not differentiate between M-level graduates and less qualified practitioners [65].

### Contextual factors

Multiple factors at the level of the individual learner, the programme of study and workplace environment were identified as potential facilitators or barriers for achieving positive M-level programme outcomes and impact.

#### Student-related context

Stress and anxiety, time management, and meeting the demand of assignments were all described as barriers for successful engagement during M-level education [39–41]. Student’s avoidance of group discussion and collaborative peers learning indicated that positive changes are less likely to occur [40]. Moreover, integrating M-level knowledge and skills were contingent on a student’s psychological preparedness for advanced level of practice [50].

#### University-related context

Students’ acceptance of scrutinising their practice was associated with supportive learning environment that offered constructive feedback [40]. These learner-centred environments were augmented by having approachable educators for a personalised feedback and support [47, 48]. However, the nature and frequency of feedback was not further examined. Moreover, consistent with principles of adult learning [67], promoting authenticity and relevance to learner’s clinical environment were seen as important to drive positive outcomes [37]. However, there were limited details of what constitutes an authentic learning environment.

#### Work-related context

It was suggested that workplace structure could limit the full integration of knowledge and skills [6, 43, 44, 50]. Whilst some graduates moved towards senior positions and advanced practice roles, others expressed a lack of enhancement because of pay and reward systems [6]. A few graduates described a lack of time, a large caseload, an uncooperative employer’s attitude, a lack of autonomous practice and a ‘poor vision’ of the NHS in accommodating their skills as barriers for continued learning [6, 41]. Having less clear job descriptions or career prospects were also documented as barriers to integration, which brought graduates into conflict with managers and colleagues [65].

### Modelling for learner’s transformation

Three studies [41, 58, 63] developed explanatory models that linked M-level programme activities and outcomes. Perry et al.’s [41] ‘knowledge acquisition’ model, which consisted of five phases, attempted to explain learner’s transformation through changing expectations and deconstructing professional knowledge followed by ‘reconstruction’ and ‘actualisation’ of M-level knowledge and skills in practice. While this model did not capture the context of transformation, Petty et al.’s [58] ‘Learning Transition Model’ highlighted the role of learner’s biography and expectations in mediating transition, in particular their reaction to the critical nature of M-level education. On the other hand, Cragg and Andrassy’s [63] evaluation of a nursing programme demonstrated an ‘evolutionary’ type of learner transformation; adding new knowledge to what graduates already knew from their undergraduate programmes.

## Discussion

This is the first methodologically rigorous review of evidence that explored taught M-level education in the healthcare context. The following is a discussion of the key findings as well as the weight of this evidence in the context of the review aims.

The review identified evidence that learners’ reactions to programme activities determined the extent of transformation. For example, whilst engagement in critical reflection drove transformative changes in practice [46], such scrutiny to one’s practice generated reactions that ranged from being defensive of their experience to being receptive to new knowledge. This gap between students’ learning dispositions and the intended outcomes of M-level education can be a source of conflict that potentially interrupts the learning process. The evidence suggests that acceptance of such scrutiny of one’s practice is associated with a supportive learner-centred environment that offers constructive feedback [40]. Such learner-centred environments were found elsewhere to promote learners’ autonomy [68–71] because of engagement in transactional relationships with their peers and educators. Moreover, learner-centred environments have the potentials to alleviate learners’ anxieties that impact learning engagement [72, 73] and therefore, achieving successful learning outcomes.

While learners’ confidence and motivation were identified as a ‘catalyst for personal growth’ [47], there is a need to further explore how extrinsic motivation at the learning site contributed to personal and professional development [73]. In accordance with Hager and Hodkinson [13], who emphasised the role of workplace structure in supporting practitioners’ learning, this review identified that learners’ motivation to maintain an advanced level of practice was dependent on workplace environment [6, 44, 50]. Graduates from several programmes expressed a sense of frustration because M-level advanced skills were not welcome within the healthcare system [6, 41, 43, 44, 65]. This potentially limits the full integration of knowledge and skills in practice, and brings graduates into conflict with managers and colleagues. For example, drawing on the experience of nursing, occupational therapy and physiotherapy educators, Gerrish et al. [74] likened graduates of nursing programmes to mavericks who did not fit comfortably into workplace cultures. They further suggested that learners’ empowerment and awakening led to conflict with managers and colleagues [74]. On the other hand, the current NHS’s post structure supports UK practitioners to working towards M-level qualification [75]. Similarly, in the context of musculoskeletal physiotherapy, Haywood et al. [76] demonstrated the positive role that physiotherapy professional bodies and employers play in supporting practitioners’ professional learning when compared with other healthcare practitioners who manage musculoskeletal conditions. Therefore, understanding motivators for professional learning would better inform M-level educators in designing effective learning environments that can cultivate learning engagement and even augment motivation. For Ryan and Deci [77], this involves understanding how the interaction between psychological and sociological aspects of motivation modify learners’ actions.

**Fig. 3:**
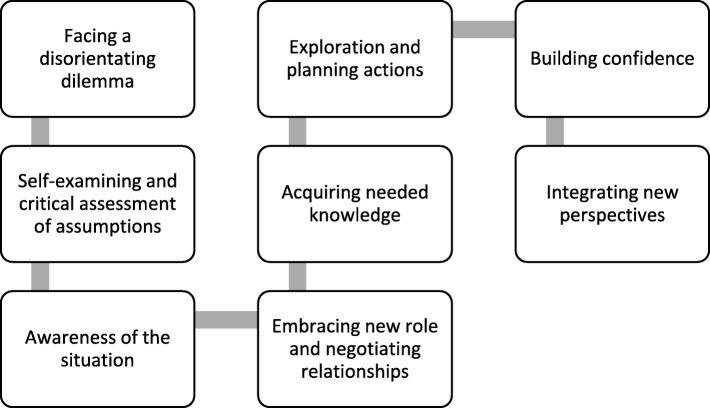
Stages of adult learners’ transformation. Adapted from Mezirow [78]

Moreover, modelling learners’ transition was described in three medium quality studies. Perry’s et al.’s [41] and Petty’s et al.’s [58] models drew on physiotherapy population and are consistent with Mezirow’s [78] stages of adult learners’ ‘revolutionary’ transition where learner’s professional identity is transformed (Fig. 3). In contrast, Cragg and Andrassy’s [64] model drew on nursing population and indicated an ‘evolutionary’ nature of transition, where learners develop their existing professional identity. This potentially explains why graduates from several nursing programmes highlighted the positive impact of their programme’s theoretical content [38, 50]. Therefore, it appears that learners’ transition is discipline-specific and influenced by workplace context. Such an interpretation is limited considering the absence of comprehensive examination of learners’ biographies.

### Weight of evidence

The overall quality of synthesised evidence demonstrated low to medium evidence across M-level pedagogy, outcomes, and impact (Table 4). This was mainly due to low to medium quality of evidence, and the inappropriate and limited relevance of included studies. With regard to methodological quality, Drennan [48] was the only researcher to use a validated assessment tool to evaluate differences in critical thinking of two nursing cohorts. While six studies [6, 38, 42, 50, 53, 62] used combined qualitative and quantitative methods of data collection, the value of this design was not clear, nor did it appear to impact the overall study conclusion. It was not clear if qualitative data were used to interpret quantitative survey; or if qualitative data facilitated the design of postal questionnaire [79]. Programme document analysis was not reported in any studies, hence losing a rich source of data related to programme structure and delivery [80]. Moreover, while qualitative-based research identified programme outcomes and impact inductively, some themes lacked theoretical saturation because of underreporting of causes, conditions, context, contingencies, consequences, and covariances [81] that would modulate changes. On the other hand, while most studies drew on graduates’ accounts, five programme evaluations drew on stakeholder data i.e. educators, managers and colleagues, adding more credibility to findings [38, 45, 49, 61, 65].

Whilst Logic Model terminologies were clearly defined and used in one study [57], terms were often ill-defined and used interchangeably across other studies; for example, ‘impact’ was used in most studies to describe the programme outcomes. Constantine and Carpenter [36], who evaluated graduates’ experience of M-level programme in musculoskeletal physiotherapy, repeatedly used terms interchangeably. Moreover, Pelletier et al. [55], who set out to evaluate the outcomes of M-level nursing programme, only reported the immediate outputs of the programme.

Where studies were retrospective in nature, the synthesised evidence offered few details linking outcomes and impact to programmes’ pedagogies, learners’ biographies and the wider context. Investigating prospective longitudinal studies would be useful to examine programme’s pedagogy and the learning context that drives change [14], capturing the frequency, type and duration of programme activities as well as making an informed judgment whether the programmes delivered learning activities as planned or not. This would usefully include a comprehensive understanding of student’s biography and learning dispositions [14], particularly in terms of understanding how learners’ biography, pre-programme clinical experience, in-service training and peer learning might contribute to outcomes and impact [82]. Moreover, it could have provided an account for how spatial (place-related) and temporal (time-related) dimensions of M-level education can influence learners’ dispositions and identity development [83, 84]. Therefore, because of the low to medium quality of included studies, the synthesised Logic Model needs to be interpreted and used with caution, and requiring further testing.

### Strengths and limitations of the review

It is suggested that a realist type of review has the potential to synthesise a programme theory that contextualises what programme components are most effective in achieving desired outcomes [85, 86]. However, since the aim of this review was to synthesise the effectiveness of a single programme in comparative contexts, the outputs of realist review may not be transferable to other programmes. A realist review would be limited since the aim is to understand how various programmes that are heterogeneous in nature (i.e. M-level health care programmes) lead to similar outcomes and impact. In this review, building an M-level programme logic model provided a more nuanced and comprehensive understanding of the complex pathways, i.e. the nuts and bolts, from conceptualisation of the programme to the application of ‘learned skills’ in the practitioners’ own environment [25, 27]. This generative theoretical explanation of change has the potential to inform planning and evaluation of M-level programmes [87].

The review has fulfilled the methodological quality criteria of evaluating and conducting systematic reviews AMSTAR [22]. In contrast to existing reviews, this review consistently used a universally-accepted Logic Model terminology of ‘output’, ‘outcome’ and ‘impact’ [88] offering a unified lens for the purpose of the synthesis. Derived conclusions are affected by the inclusion of low-medium quality studies, where aims are not fully aligned to the objectives of this study. Studies involving ‘residency’ and ‘fellowship programmes’ were not included. These programmes can be equivalent to M-level education, yet they are not based in higher education settings.

## Conclusion

Findings from this methodologically rigorous review underpin two key points. Firstly, multiple positive outcomes and areas of impact are reported in M-level healthcare education. This synthesised evidence was derived from retrospective studies in which a single method of data collection was used, thus, limiting the generalisability and transferability of findings. Secondly, although the link between programme pedagogy, context and outcomes was underreported, drawing on contemporary learning theories such as participatory learning [13, 14] is believed to produce the intended learning outcomes. Future research need to examine, through a longitudinal empirical study, learners’ dispositions prior, during and after engagement in M-level healthcare education as well as the influence of various contexts to bridge this gap in evidence.

## Additional files

Additional file 1: Excluded Studies. (DOCX 25 kb)

Additional file 2: Studies that reported key didactic features of the evaluated M-level programmes. (DOCX 29 kb)

## Data Availability

The datasets analysed in this review are available from the corresponding author on reasonable request.
