# Supplementary material for: Exploring taught masters education for healthcare practitioners: a systematic review of literature
Source: BMC Med Educ. 2019 Sep 5;19:340. doi: 10.1186/s12909-019-1768-7 (PMC6729035; doi:10.1186/s12909-019-1768-7)
Supplement: Supplementary file 1 — Excluded Studies. (DOCX 25 kb) [file 12909_2019_1768_MOESM1_ESM.docx]

**Additional file 1: Excluded Studies.**

| Reason for exclusion | Total Number | References |
| --- | --- | --- |
| Potential impact | 10 | (Beeston et al., 1998); (Caldwell, 2001); (Darby, 2009); (Finocchio et al., 2003); (Gerrish et al., 2003); (Hardcastle, 2008); (Gosling, 1997); (Gosling, 1999); (Rushton and Lindsay, 2008); (Brody et al., 2012) |
| Online master's degree | 2 | (Baker and Lewis, 2007); (Richardson et al., 2008) |
| Off-campus studies | 3 | (Bethune and Jackling, 1997); (Davis et al., 2004); (Schattner et al., 2007) |
| Combined programmes evaluation | 2 | (Boore, 1996); (Hardwick and Jordan, 2002); |
| None master-level PG programme evaluation | 4 | (Stacey et al., 2010); (Ikai et al., 2012); (Glaze, 2001); (Rushton and Lindsay, 2010); (Stellman et al., 2008) |
| No full text | 2 | (Pelletier et al., 1998); (Ruth et al., 2006) |
| Combined PG and Non-PG cohort | 1 | Johnson & Copnell (2002) |
| Other non-healthcare master | 6 | (Balogh, 2012); (Spigulis, 2000); (Edgar and Hyde, 2005); (Filizetti, 2003); (Fletcher, 2005); (Wilson and Wen, 2000) |
| Clearly not meeting inclusion criteria | 40 | E.g. (Dehn, 2007); (Evans et al., 2006); (Fraser and Titherington, 1991); (Hooker, 2009); |
| Poor quality of evidence Using MAAT | 4 | Baron et al. (2001); Gill et al. (2005); Harris et al. (2008); Plugge and Cole (2011) |
