# Supplementary material for: Exploring taught masters education for healthcare practitioners: a systematic review of literature
Source: BMC Med Educ. 2019 Sep 5;19:340. doi: 10.1186/s12909-019-1768-7 (PMC6729035; doi:10.1186/s12909-019-1768-7)
Supplement: Supplementary file 2 — Studies that reported key didactic features of the evaluated M-level programmes. (DOCX 29 kb) [file 12909_2019_1768_MOESM2_ESM.docx]

**Additional file 2: Studies that reported key didactic features of the evaluated M-level programmes**

| Name of programme | Programme philosophy and main pedagogy |
| --- | --- |
| MSc Neuromusculoskeletal Physiotherapy [39, 40] | - Theoretical content - Mentored clinical practice - Direct observation and critical feedback - Critical classroom discussion and evaluation of practice knowledge - Challenging clinical reasoning problems |
| King's MSc in General Practice [42, 43] | - Forming learning groups - Seminar discussion of preselected readings - Closing theory-practice gaps - Personalising learning experience - Ongoing assessment |
| MSc Physiotherapy & M.Sc. Manipulative Physiotherapy [44] | - Critical thinking activities - Self-direction learning |
| Orthodontic problem-based postgraduate programme [49] | - Problem-based learning |
| MSc in General Practice [50] | - learner-centred approach - Peer support and shared learning - Work in groups - Construct practice development plans - Reflection - Approaching tutors for feedback - Role-play and modelling |
| Geriatrics Nursing [52] | - Sharing experiences - Mentored clinical experience |
| MSc in Nursing and Health Studies [56] | - Problem solving environment - Learner-centred pedagogy - Flexibility of delivery - Personalised feedback and support |
| Critical Care Graduate Diploma [62] | - Theoretical content - Collaborative interaction - Reflection on experience - Clinical placement |
| Advanced Neonatal Nurse Practitioner programme [63] | - Theoretical content |
